# Mitochondrial Phenotypes in Parkinson’s Diseases—A Focus on Human iPSC-Derived Dopaminergic Neurons

**DOI:** 10.3390/cells10123436

**Published:** 2021-12-07

**Authors:** Leonie M. Heger, Rachel M. Wise, J. Tabitha Hees, Angelika B. Harbauer, Lena F. Burbulla

**Affiliations:** 1 Biomedical Center (BMC), Division of Metabolic Biochemistry, Faculty of Medicine, Ludwig-Maximilians-Universität München, 81337 Munich, Germany; leonie.heger@med.uni-muenchen.de (L.M.H.); rachel.wise@med.uni-muenchen.de (R.M.W.); 2 Max Planck Institute of Neurobiology, 82152 Martinsried, Germany; thees@neuro.mpg.de (J.T.H.); angelika.harbauer@neuro.mpg.de (A.B.H.); 3 Institute of Neuronal Cell Biology, Technical University of Munich, 80333 Munich, Germany; 4 Munich Cluster for Systems Neurology (SyNergy), 81337 Munich, Germany; 5 German Center for Neurodegenerative Diseases (DZNE), 81337 Munich, Germany

**Keywords:** Parkinson’s disease, iPSC, mitochondria, dopaminergic neurons

## Abstract

Established disease models have helped unravel the mechanistic underpinnings of pathological phenotypes in Parkinson’s disease (PD), the second most common neurodegenerative disorder. However, these discoveries have been limited to relatively simple cellular systems and animal models, which typically manifest with incomplete or imperfect recapitulation of disease phenotypes. The advent of induced pluripotent stem cells (iPSCs) has provided a powerful scientific tool for investigating the underlying molecular mechanisms of both familial and sporadic PD within disease-relevant cell types and patient-specific genetic backgrounds. Overwhelming evidence supports mitochondrial dysfunction as a central feature in PD pathophysiology, and iPSC-based neuronal models have expanded our understanding of mitochondrial dynamics in the development and progression of this devastating disorder. The present review provides a comprehensive assessment of mitochondrial phenotypes reported in iPSC-derived neurons generated from PD patients’ somatic cells, with an emphasis on the role of mitochondrial respiration, morphology, and trafficking, as well as mitophagy and calcium handling in health and disease. Furthermore, we summarize the distinguishing characteristics of vulnerable midbrain dopaminergic neurons in PD and report the unique advantages and challenges of iPSC disease modeling at present, and for future mechanistic and therapeutic applications.

## 1. Introduction

The brain is responsible for nearly 20% of the body’s energy consumption, which is remarkable considering that it represents a mere 2% of total body mass [1]. This is primarily driven by the large number of neurons, with energetically-demanding processes including synaptic activity making them highly dependent on mitochondrial function [2,3]. The mitochondrial process of oxidative phosphorylation (OXPHOS) generates energy in the form of adenosine triphosphate (ATP) molecules necessary for cellular function and survival, but also reactive oxygen species (ROS) which can, if not properly scavenged, cause DNA damage, protein modifications and other deleterious changes within the cell [4]. Mitochondria also regulate neuronal health and function by buffering calcium (Ca^2+^) transients from both extracellular sources and intracellular storage organelles like the endoplasmic reticulum (ER), protecting against Ca^2+^ overload and encoding these complex electrochemical signals into coordinated neurotransmission [5]. To accomplish this and to meet the energy demands of distinct neuronal microdomains, mitochondrial transport along axonal microtubules is tightly regulated in both retrograde and anterograde directions. Mitochondrial fitness is crucial for the abovementioned functions and cellular survival; thus, extensive quality control mechanisms have evolved to maintain healthy mitochondrial networks and eliminate damaged or aged organelles. Through frequent fusion and fission events, mitochondria compensate for accumulation of mtDNA damage, preserving healthy networks and isolating damaged fragments for elimination. With significant damage, mitochondria undergo selective autophagic degradation termed mitophagy, preventing the build-up of potentially neurotoxic organelles. 

Mitochondrial dysfunction can have dire consequences for long-lived, postmitotic neurons, causing rampant oxidative stress, overwhelming ROS burden, metabolic dyshomeostasis, and impaired Ca^2+^ buffering, ultimately culminating in impaired cellular function and potentially triggering apoptotic pathways [6]. Therefore, mitochondrial dysfunction is increasingly proposed as a key aspect of neurodegenerative diseases such as Parkinson’s disease (PD). The study of neurodegeneration and related pathologies has previously been limited to post-mortem brain tissue, simple cellular models, and transgenic, pharmacological, or toxin-induced animal models of disease [7,8,9]. Each model system has yielded valuable insight into pathogenic mechanisms, but translational progress has been hindered by insufficient recapitulation of human disease characteristics in animals or non-neuronal cells. The emergence of induced pluripotent stem cell (iPSC) technology has revolutionized the field of neurodegenerative disease modeling, allowing the investigation of molecular mechanisms and therapeutic strategies in relevant human cell types derived from the patients’ own somatic cells. Thus, in this article we present a comprehensive review of studies on mitochondrial phenotypes in iPSC-derived neuronal models of PD, and how this may contribute to the disease-specific degeneration of susceptible neuronal populations in PD. 

## 2. Selective Neuron Vulnerability 

After Alzheimer’s disease, PD is the second-most common neurodegenerative disease, affecting approximately 1% of the population over the age of 60 and up to 3% of the population over 80 [10]. Patients typically present with early non-motor symptoms including depression and anxiety, speech problems, and sleep disturbances, and later develop postural imbalance, bradykinesia, muscle rigidity, and resting tremors [11]. Most PD cases are sporadic and of unknown etiology, while roughly 5–10% are due to confirmed genetic causes. These genetic, or familial, forms of PD result from a variety of autosomal dominant (*LRRK2, GBA1, SNCA, VPS35*) and autosomal recessive (*PINK1, Parkin, DJ-1, ATP13A2, PLA2G6, VPS13C*) mutations which typically present with earlier onset than sporadic PD [12]. Genetic variability in *LRRK2* (leucine-rich repeat kinase 2) is the most common genetic cause of sporadic and familial PD with the G2019S mutation being the most common genetic determinant of PD identified to date [13]. The strongest genetic risk factor is genetic variation in the *GBA1* gene encoding for the lysosomal enzyme glucocerebrosidase. Whereas homozygous variants in *GBA1* are implicated in autosomal-recessive Gaucher’s disease, heterozygous carriers of *GBA1* mutations were shown to have a four- to five-fold increased risk of developing PD [14]. Multiplications (duplications, triplications) of the *SNCA* gene encoding for alpha-synuclein (α-syn), a natively unfolded protein predominantly localized to the presynaptic terminal, have been shown to cause autosomal dominant PD as well [15,16]. Despite the wide etiological spectrum, PD patients typically display a common set of pathological hallmarks including intra-neuronal inclusions, or Lewy bodies, in surviving cells of all affected brain regions and loss of dopaminergic neurons within the substantia nigra pars compacta (SNpc). Lewy bodies are composed of a variety of proteins with the majority being abnormally aggregated α-syn [17]. The process of Lewy body formation and the interaction with α-syn induces mitochondrial dysfunction, though the exact underlying mechanisms are not well understood [18].

While other neuronal populations are also lost early in PD pathogenesis and are purported to underlie the non-motor symptoms that precede typical PD diagnosis [19], it is the loss of dopaminergic SNpc neurons which results in depleted dopaminergic innervation of the striatum and the characteristic motor symptoms commonly observed in PD patients. It remains unclear why this neuronal subpopulation is selectively vulnerable, especially given that dopaminergic neurons of the neighbouring ventral tegmental area (VTA) are more resilient [20]. Nigral dopaminergic neurons demonstrate key differences with those in the VTA, which may explain some aspects of their unique susceptibility in PD, most notable is their long unmyelinated axons with complex arborization and high mitochondrial density [21,22,23], and their autonomous pacemaking activity accompanied by large Ca^2+^ oscillations and inherently low Ca^2+^ buffering capacity relative to those in the VTA [24,25,26].

## 3. Parkinson’s Disease Modeling Using Human iPSC Technology

Like other neurodegenerative disorders, PD remains a sizeable clinical challenge with no cure and few, symptomatic treatment options. Disease models are indispensable tools for uncovering pathological mechanisms of neurodegeneration and development of novel therapeutics. The implementation of increasingly sophisticated animal models has led to greater understanding of the molecular and cellular mechanisms driving disease processes and has provided in vivo systems for assessment of innovative intervention strategies. Unfortunately, many therapeutic efforts developed in animals fall short in clinical trials. To overcome these persistent translational challenges, there is a critical need to generate disease models that more faithfully recapitulate human pathophysiology.

With the inaccessibility of primary neurons from human patients, iPSC technology provides an unprecedented tool to study the most affected cell populations from individuals with a verified pathogenic genetic background [27,28]. iPSCs can self-renew and differentiate into various cell lineages, offering a potentially unlimited platform to examine diverse cellular phenotypes and interactions. iPSC-derived platforms employed as in vitro disease models have added invaluable insight into PD pathogenesis by manifesting characteristics commonly found in human PD pathology [29,30]. This foundation allows for the investigation of phenotype-correcting effects of novel compounds on patient-specific iPSC-derived model systems, providing a valuable pipeline for the identification and preclinical development of therapeutic agents [31]. Since the advent of iPSC technology, enormous strides have been made in stem cell biology and regenerative medicine. Moreover, differentiation protocols and quality control criteria are regularly updated and improved, making iPSCs a rapidly evolving technology for physiologically-relevant in vitro modeling of human disease. These advances have generated protocols which routinely yield neuronal cultures comprised of 50–70% midbrain dopaminergic neurons, supporting the investigation of the physiology and function of this unique neuronal subset [32,33,34]. Given the numerous advantages of iPSCs over alternative models—including their human origin, the ability to be differentiated into many cell types, particularly those with limited accessibility, and the therapeutic potential of patient-specific iPSCs in personalized medicine—iPSC-based platforms present a unique opportunity for exploring disease pathogenesis and identifying novel drug targets for debilitating neurodegenerative diseases. In recent years, a number of research groups have successfully generated iPSCs from patients with sporadic or familial PD, and mounting evidence has revealed mitochondrial disturbances as a significant, and potentially shared, contributing factor to pathology, highlighting the importance of this organelle for neuronal health and disease.

Despite the many advantages, there are also limitations to the use of iPSC-derived neurons as a model system. To investigate pathogenic mechanisms relevant to PD, it is critical to establish mature dopaminergic neuronal cultures. While some indicators of dopaminergic lineage are present by 35 days in culture including tyrosine hydroxylase (TH) expression and dopamine synthesis and release, reports have shown that functional properties of iPSC-derived dopaminergic neurons change dynamically between 6 and 10 weeks in culture. In fact, spontaneous calcium oscillations and autonomous pacemaking activity are not fully established until roughly 70 days in culture [35]. In support of this, one group reported greater expression of immature, and lesser expression of mature, dopamine neuron transcripts at 50 days in culture compared to their primary human midbrain counterparts [36]. Thus, it is critical to allow sufficient time in culture for these model neurons to reach maturity and enable more accurate comparisons to the adult human brain. iPSC-derived neurons have found widespread use in early stage drug discovery pipelines, have been successfully used to test the impact of compounds with known modes of action, and have opened new avenues for evaluating drug repurposing strategies [37]. However, the sole use of two-dimensional monolayer cultures is unlikely to recapitulate the complexity and function of three-dimensional (3D) in vivo neural circuits, and do not take into account the unique challenge of the blood-brain barrier or the whole organism [38]. In contrast to single cell-type cultures, organoids consist of multiple cell types that self-organize spatially and can display enhanced cellular maturation and functionality, possibly due to the more physiological 3D niche environment [39]. Moreover, co-culture systems of neurons and glia in bioengineered neural circuit model systems are needed to fully understand non-cell autonomous determinants of PD disease progression. Astrocytes were previously considered merely metabolic support for neurons, but recent reports have revealed their active participation in regulating neuronal activity, the formation of neural networks, recycling of neurotransmitters, providing neurotrophic factors and detoxification [40,41,42].Another pitfall in iPSC modeling is the lack of standardization among differentiation protocols leading to variability in culture quality, maturation status, and experimental outcomes and data interpretation. The importance of this was shown in a study from Chung and coworkers which demonstrated the recapitulation of disease-related phenotypes in PD patient iPSC-derived midbrain dopamine neurons generated using a floor-plate-based protocol, but a lack of observable phenotypes using a neural-rosette-based directed differentiation strategy [43]. Another major confound is donor-to-donor variability, particularly among patients carrying identical genetic risk variants. To minimize this challenge, new technologies such as clustered regularly interspaced short palindromic repeats (CRISPR) genome editing can be combined with human iPSC model systems to generate isogenic mutant and control cell lines, facilitating the delineation of pathogenicity of variants of uncertain significance [44].

While iPSCs hold great promise for PD, there are still scientific and clinical challenges that must be addressed to improve the translational potential for development of novel therapeutics. Nevertheless, iPSC technology has significantly changed the field of neurodegenerative research and will be instrumental in improving our understanding of mechanistic pathways in the pathology of PD.

## 4. Discoveries of Mitochondria-Specific Phenotypes in iPSC Models of PD

### 4.1. Mitochondrial Respiration and Membrane Integrity

Mitochondrial ATP synthesis is accomplished through the combined activity of the tricarboxylic acid cycle (TCA) (also known as the Krebs or citric acid cycle). This series of chemical reactions occurs in the mitochondrial matrix, feeding high energy NADH and FADH_2_ into the electron transport chain (ETC) in the inner mitochondrial membrane (IMM) to facilitate OXPHOS, the primary source of energy production in neurons. The flow of electrons from NADH or FADH_2_ to O_2_ through the ETC protein complexes I-V facilitates the pumping of protons out of the mitochondrial matrix. The resultant uneven distribution of protons generates a pH gradient and a transmembrane potential (ΔΨm) that creates a proton force and drives ATP synthesis through the activity of complex V, ATP synthase [45,46]. OXPHOS also generates potentially toxic byproducts in the form of ROS, which, if not tightly regulated by antioxidant defenses, can disrupt mitochondrial physiology, damage DNA, modify protein functions, cause lipid peroxidation, and negatively influence signaling pathways including transcriptional regulation [47,48]. Hence, all mitochondrial processes can be impacted by unbalanced ROS regulation and may thus represent a common pathway/potential starting point for neurodegenerative diseases [49,50,51]. Neurons are characterized by a high metabolic demand and require reliable ATP synthesis for diverse neuro-communication functions including maintenance of resting membrane potential, neurotransmitter synthesis and release [52], thus, disruption of this process is particularly detrimental to neuron health and survival. Consequently, determination of mitochondrial respiration defects, namely expression and function of ETC protein complexes, OXPHOS dynamics, changes to ΔΨm, and ATP synthesis capacity have become a major focus of PD research. 

**Figure 1:**
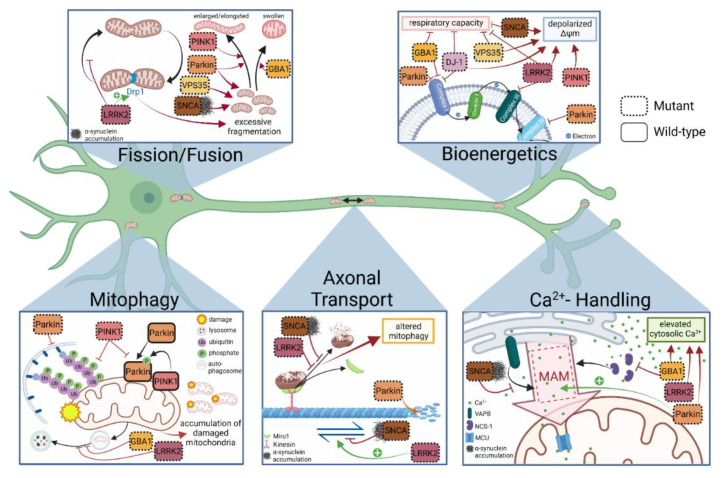
***Bioenergetics*:***GBA1* and *Parkin* deficiency, with mutant *Parkin* also being involved in suppression of complex IV proteins. Furthermore, a major bioenergetic burden has been reported in genetic PD neurons with mutations in *DJ-1, GBA1, LRRK2, VPS35* and *SNCA* to result in reduced mitochondrial respiration capacity, or in neurons with mutant *LRRK2*, *VPS35*, *SNCA* and *PINK1* to suffer from reduced mitochondrial membrane potential (ΔΨm). ***Fission/Fusion***: The balance of fission and fusion has been shown be disrupted in several iPSC-derived models of genetic PD with mutant *PINK1* and *Parkin* leading to enlarged/elongated organelles, and mutant *GBA1* to rather swollen mitochondrial structures. Excessive mitochondrial fragmentation has been demonstrated in mutant *Parkin* and *VPS35* neurons, as well as in neurons from *SNCA* and *LRRK2* patients, a phenotype that is suggested to be associated with increased Drp1 activity in the latter. ***Axonal transport***: Aberrant α-syn expression was shown to interfere with anterograde axonal transport, while mutant *LRRK2* leads to enhanced mitochondrial motility in human neurons. Alterations of both proteins have also been shown to be involved in Miro1 kinetics by delaying Miro1’s removal from mitochondria, thereby interfering with proper mitochondrial clearance (mitophagy) under conditions of mitochondrial depolarization. Additionally, destruction of microtubules themselves has been reported in *Parkin* patient neurons. ***Mitophagy***: PINK1/Parkin-dependent mitophagy has been well characterized, hence, not surprisingly, mutant *PINK1* and *Parkin* patient neurons demonstrate impaired mitophagic flux, partially based on studies reporting reduced levels of phosphorylated ubiquitin (Ser65) and impaired recruitment of Parkin to mitochondria upon mitochondrial depolarization. Both *LRRK2* and *GBA1* mutations have been shown to interfere with autophagosome (AP) to lysosome transport or mitochondrial-lysosomal colocalization, respectively. ***Ca^2+^ handling***: Disrupted association between ER and mitochondria for regulation of Ca^2+^ transfer at MAM sites is a shared phenotype among *GBA1*, *Parkin* and *SNCA* PD mutant neurons, reported to show altered Ca^2+^ handling. While *Parkin* mutant neurons show an increased ER-mitochondria association resulting in excessive uptake of Ca^2+^ into mitochondria, both *SNCA* triplication neurons and *GBA1* mutant neurons suffer from reduced Ca^2+^ transfer into mitochondria due to (a) interference of α-syn with the ER-associated protein VAPB (*SNCA* triplication), and (b) reduced levels of neuronal calcium sensor-1 (NCS-1) (*GBA1* mutant neurons) that facilitates MAM formation under normal conditions.

Several lines of evidence have emerged linking deficient ETC complex activity and dopaminergic degeneration. Early examinations of human post-mortem SNpc tissue demonstrated decreased complex I activity [53,54], and investigations in rodent neurons yielded insight into possible mechanism by showing that interactions of pathological α-syn oligomers, but not monomers, disrupted complex I and complex V of the ETC resulting in impaired respiration [55]. The connection between dysfunction of complex I and PD emerged in the mid-1980s, when young adults developed severe and irreversible parkinsonism shortly after injecting a new designer street drug contaminated with the compound MPTP (1-methyl-4-phenyl-1,2,3,6-tetrahydropyridine), a potent neurotoxin targeting mitochondria in dopaminergic neurons within the SNpc [56,57]. MPTP’s active metabolite MPP+ (N-methyl-phenylpyridinium ion) is a known inhibitor of complex I of the ETC [58,59], causing decreased production of ATP [57] and elevated generation of ROS [60]. Interestingly, deficient complex I activity has been confirmed in animal and non-neuronal models of genetic PD, i.e., *Parkin* [61], *PINK1* [62], and *DJ-1* [63,64,65,66], with the latter being shown to be able to directly bind to subunits of complex I [63]. In iPSC-derived neuronal models to date, impaired ETC complex I activity has been described in *Parkin* mutant [67] and *GBA1* mutant neurons [68], while LRRK2 G2019S mutant neurons displayed reduced complex III expression [69] (Figure 1). 

However, this finding has been debated for *Parkin* mutant neurons, as a recent proteomic analysis reported unchanged complex I levels but rather specific suppression of complex IV proteins [70]. This did not correlate with impairments in mitochondrial respiration, but rather impaired glycolysis and lactate metabolism which was associated with worsened neuronal viability. This finding may be noteworthy as astrocyte-derived lactate has proven neuroprotective potential in several pathological conditions [71,72]. Interestingly, the mitochondrial protein stomatin-like protein 2 (SLP-2) interacts with both Parkin and cardiolipin and promotes the assembly of respiratory chain proteins, and overexpression of this protein in *Parkin* mutant models rescued complex I function in iPSC-derived neurons [67]. 

While different PD genotypes appear to impact distinct components of the respiratory machinery, the resulting mitochondrial respiratory defects show many similarities. As determined by live-cell assessment of oxygen consumption rates (OCR), basal, maximal, and spare mitochondrial respiratory capacity is reduced in both *VPS35* [73] and *GBA1* mutant iPSC-derived dopaminergic neurons [68]. In a direct comparison of LRRK2 G2019S PD patient iPSC-derived dopaminergic, glutamatergic, and peripheral sensory neurons [69], only the central neuronal subtypes displayed impaired ATP-linked, maximal, and spare respiratory capacity, with intact basal mitochondrial respiration. However, deficits in basal respiration have been reported in LRRK2 G2019S and LRRK2 R1441C patient dopaminergic neurons [74]. Overall, this suggests that neurons of the central, but not the peripheral, nervous system are more susceptible to mitochondrial deficits. It was further demonstrated that only dopaminergic neurons had reduced ATP and ADP pools, which may have been associated with weakened PGC1α-dependent mitochondrial biogenesis [69], providing another potential mechanism contributing to the vulnerability of dopamine neurons. Interestingly, some of these impairments were also reported in LRRK2 G2019S neural stem cells (NSCs), indicating some pathological phenotypes may be neurodevelopmental [75]. In *SNCA* triplication PD patient iPSC-derived neural progenitor cells (NPCs), as well as CRISPR-induced *SNCA* mutant (A30P and A53T) neuroepithelial-like stem cells (NESCs) and patient-derived dopaminergic neurons increased α-syn oligomer burden was correlated with suppressed basal, ATP-linked, and spare respiratory capacity [76,77,78], as well as decreased ATP production which could be restored with *SNCA* knockdown [77]. Studies using mature patient-derived iPSC dopamine neurons from PD patients carrying either the A53T α-syn mutation or a triplication of the *SNCA* locus reported reduced maximal and spare mitochondrial respiration capacity and ATP production, with conflicting results on impaired basal respiration [79,80]. These investigations further demonstrate that despite a spectrum of mutation-specific impact on cellular bioenergetics, up-to-date investigations of PD neurons share the common hallmark of repressed mitochondrial respiratory function at baseline and/or in response to metabolic stress.

Proper functioning of the ETC generates and maintains the ΔΨm to facilitate ATP synthase activity and maintain organellar fitness. Sustained loss of membrane integrity can lead to impaired OXPHOS, reduced ATP pools, release of cytochrome c into the cytosol, and triggering of cell death pathways. In iPSC-based neuronal models of PD, NPCs [77], cortical neurons [55], and dopaminergic neurons [81] from *SNCA* mutant or triplication PD patients, as well as NESCs from LRRK2 G2019S PD patients [75] and dopaminergic neurons from VPS35 [73], displayed reduced ΔΨm. In iPSC-derived neurons from PD patients with *PINK1* mutations, ΔΨm and ATP content was lowered [82,83], consistent with an earlier report of Morais and coworkers which demonstrated in animal models of diminished PINK1 function that impaired complex I function resulted in mitochondrial depolarization, reduced ATP synthesis, and increased sensitivity to apoptotic stress [62]. Interestingly, iPSC-derived dopaminergic neurons from PD patients with heterozygous *GBA1* mutations (N370S, L444P, and RecNcil) showed no alterations to ΔΨm, despite substantial respiratory defects and elevated mitochondrial ROS generation [68]. Dysfunction of the ETC, compromised ΔΨm, and superoxide and ROS by-products of OXPHOS are strongly connected with the generation of mitochondrial oxidant stress in both *DJ-1* knockout animals [25] and iPSC-derived dopaminergic neurons from PD patients with a loss-of-protein mutation in DJ-1 [34], as was demonstrated using a redox-sensitive variant of green fluorescent protein (roGFP) with a mitochondrial matrix targeting sequence. Interestingly, the latter study found a time-dependent toxic cascade starting with mitochondrial oxidant stress and reduced basal respiration leading to accumulation of α-syn and lysosomal dysfunction in DJ-1 mutant PD neurons and identified oxidized dopamine derivates as the mediators of this pathogenic sequence.

Taken together, the use of iPSC-derived patient neurons has revealed that while mitochondrial respiration may represent a shared pathology across sporadic and genetic forms of PD, the manner and extent to which these interconnected processes are influenced by accumulated α-syn and diverse genetic backgrounds varies and will therefore require more complex and personalized targeting for therapeutic development in future. 

### 4.2. Mitochondrial Fusion, Fission and Morphology 

Mitochondria are highly dynamic organelles that are able to adapt to different cellular states by changing their morphology [84]. To maintain network integrity, mitochondria utilize both fission and fusion to sustain fitness levels. These balanced transitions preserve organellar function and allow for versatile responses to cellular needs by adapting the network to nutrient availability or optimal metabolic state of the cell [85,86,87].

Mitochondrial fission is the division of a single organelle into two or more independent structures. This process is mediated by two proteins: dynamin-related protein 1 (Drp1) as part of the GTPase family and human fission 1 (hFis1) located in the outer mitochondrial membrane (OMM) which coordinate membrane scission [88], while the IMM constriction is independently regulated by calcium influx [86,89]. The two-step process of fusion results in the union of two mitochondria and both their inner and outer membranes. First, trans-dimerization between homologous proteins mitofusion 1 and 2 (MFN1/2) leads to formation of a curved complex which tethers both membranes together, followed by the GTPase activity which allows fusion of the OMM by initiating conformational change [90]. Subsequent fusion of the IMM is dependent on membrane potential and the GTPase optic atrophy protein 1 (OPA1) [91]. The regulation of mitochondrial size and length is very important for their subcellular localization and overall number. As neurons are post-mitotic and do not undergo further cell division, fusion and fission enable the exchange of mitochondrial contents and equal distribution of metabolites [92,93,94]. This ability to continuously adjust to the energy demands in distinct cellular compartments is particularly important for neurons which possess a highly polarized structure with areas of unique metabolic requirements and different mitochondrial distributions. Furthermore, fission is a critical prerequisite for isolating depolarized mitochondria for the selective autophagic process termed mitophagy and lysosomal-based degradation [95].

Abnormalities in mitochondrial fission or fusion have been suggested to occur as early events during the pathogenesis of many neurological disease states [93]. However, the exact mechanism of altered mitochondrial dynamics and morphology associated with mutations in various PD-associated genes is controversial. As an example, while *Parkin* mutations in Drosophila lead to abnormal mitochondrial morphology [96], mitochondria in *Parkin* knockout mice display no gross morphological abnormalities [97]. Interestingly, different *Parkin* patient models also differ in their representation of mitochondrial alterations, i.e., iPSC-derived neurons, but not fibroblasts or iPSCs, from patients with *Parkin* mutations present with abnormal or enlarged mitochondria [98]. This was independently confirmed in *Parkin* patient iPSC-derived dopamine neurons, with similar phenotypes observed in *PINK1* patient neurons as well [43]. However, no altered mitochondrial morphology was observed in homozygous PINK1 knockout human dopaminergic neurons [99]. An increase in the numbers of elongated mitochondria in *Parkin* patient neurons was also reported by Bogetofte and colleagues [70]. Interestingly, their mitochondria-specific proteomic analysis revealed a large number of dysregulated mitochondrial proteins in Parkin knockout iPSC-derived dopaminergic neurons compared to isogenic controls. Conversely, *Parkin* patient-derived NPCs showed a subtle, but significant elevation in mitochondrial fragmentation that dose-dependently increased in severity with copper exposure, a known environmental risk factor for PD [100]. A recent study using Parkin KO iPSC-derived neurons elegantly showed that smaller and less functional mitochondria were present in dopaminergic, but absent in non-dopaminergic neurons using a tyrosine hydroxylase reporter cassette and correlative light electron microscopy [101]. This suggests that dopaminergic neurons possess intrinsic physiological differences, making them particularly vulnerable compared to other types of neurons. Moreover, iPSC-derived NESCs [75] and dopaminergic neurons [102] from LRRK2 G2019S patients with hyperactive LRRK2 displayed excessive mitochondrial fission and more fragmented organelles. Mechanistically, this may be linked with LRRK2-mediated increase in Drp1 activity, as pharmacological or genetic modification of Drp1 altered its translocation to the OMM and restored mitochondrial fission, thereby providing neuroprotective effects [102]. Finally, smaller and more fragmented mitochondria were observed in mice with dopamine neuron-specific deletion of VPS35, which was correlated with downregulated MFN2 expression [103], and in iPSC-derived dopaminergic neurons from PD patients with the VPS35 D620N mutation, which also demonstrated decreased network connectivity [73].

Direct association between mitochondrial membranes and α-syn lead to mitochondrial fission and disorganized cristae independent of Drp1, as demonstrated in α-syn-overexpressing HeLa cells and murine midbrain neurons [104]. While patient-derived NPCs from α-syn triplication patients did not show differences in mitochondrial shape [77], the phenotype of fragmented mitochondria associated with altered α-syn was confirmed in iPSC-derived dopaminergic neurons from patients with *SNCA* mutation (A53T) [105]. Interestingly, cardiolipin translocation to the OMM and the subsequent loss of mitochondrial surface charge preceded this mitochondrial fragmentation pathology in *SNCA* mutant neurons, revealing a potential novel mechanism. Altered cristae morphology and increased mitochondrial diameter was also observed in iPSC-derived dopaminergic neurons from PD patients carrying heterozygous *GBA1* mutations (N370S, L444P, and RecNcil), and were concomitant with dysregulated levels of mitochondrial shaping proteins Drp1, OPA1, and Mfn1 [68]. 

The regulation of mitochondrial morphology is a complex balance between fusion- and fission-related proteins and situation-dependent distribution events. The exact link between altered mitochondrial morphology and the development of neurodegenerative diseases like PD is not yet clear, and will require more studies on disease-relevant cell types to shed light on the link between the actual structure of mitochondria and pathological processes in neurons.

### 4.3. Axonal Transport of Mitochondria

Mitochondrial transport allows cells to respond to regional changes in metabolism and protect mitochondrial integrity. In neurons, the location of mitochondria is particularly important due to the unique architecture of highly branched dendrites and long, complex axons which can extend up to one meter, as is the case for some human motor neurons. Mitochondrial proteins are primarily synthesized in the soma and therefore must be transported to distal sites to provide the energy required to maintain cellular function. Axonal transport of mitochondria happens bidirectionally and is almost entirely microtubule-dependent. Anterograde transport supplies distal axons with organelles, proteins and lipids required for maintaining presynaptic activity, while retrograde transport removes aged components that are primarily degraded and recycled back in the soma. Mitochondrial cargo is transported via the motor protein kinesin-1 [106] that binds to the membrane anchors Miro1/2, small Rho GTPases, and their motor adaptors Trak1+2 (Milton) to control proper anterograde trafficking to the synaptic compartment [107,108,109]. Retrograde transport of mitochondria uses the major motor protein dynein with its activator dynactin, a highly conserved multiprotein complex essential for normal neuronal function [110]. Perturbations in either form of axonal transport can be detrimental to neuronal health, causing metabolic dyshomeostasis, oxidative stress, and even cell death.

While various model systems have already contributed valuable insight into axonal transport of mitochondria, human iPSC-derived platforms offer the unique opportunity to disentangle this process in a human, potentially patient-derived system over prolonged periods of time in culture. Indeed, one report using wild-type iPSC-derived dopaminergic neurons “aged” in culture revealed a time-dependent increase in immobile mitochondria and reduction in anterograde transport from 40 to 100 days [111]. This may point to a general decline in mitochondrial trafficking during “aging” of human neurons with eventual consequences on neuronal viability. 

Disturbed mitochondrial motility as a major factor in the development of PD is a longstanding debate. Animal studies have established that early signs of the disease begin in synaptic terminals, suggesting dysregulation of axonal transport of proteins and organelles, including ATP-generating mitochondria, towards the synapse as an early event in PD pathogenesis [112,113]. In recent years, data from human and mouse models suggest that dysfunction of the microtubule network contributes to the pathogenesis of PD, however, the extent of altered microtubules in PD patients is not yet clear. Patient fibroblast studies revealed microtubule destabilization in the context of PD pathogenesis [114], and initial studies in iPSC-derived neuronal cells from PD patients carrying mutations in *LRRK2* confirmed altered mitochondrial mobility in the axon [69,74]. Fragmentation of microtubules also preceded mitochondrial transport defects in iPSC-derived dopamine neurons from PD patients with *Parkin* mutations, as well as in a knockout mouse model [115]. 

Further studies address the effects of PD-associated mutations on mitochondrial motility by modifying proteins that regulate mitochondrial trafficking in disease-relevant models. Miro1, the primary regulator of mitochondrial transport in both axons and dendrites, is particularly relevant to PD. In a Miro1 knockout mouse model [116], loss of protein function slowed mitochondrial trafficking along microtubules. In line with this, PD patient iPSC-derived neurons with mutant *Miro1* (Miro-R272Q) exhibited decreased mitochondrial velocity when compared with control neurons [117]. Further, α-syn oligomerization in different iPSC-derived neuronal models of aberrant α-syn expression (duplication, mutant E46K, oligomer-prone E57K a-syn mutant) lead to a reduction of Miro1 and kinesin light chain 1 (KCL1) proteins in neurites resulting in disrupted anterograde axonal transport of mitochondria [118]. As an OMM protein, Miro1 anchors mitochondria to microtubule motors. To eliminate damaged mitochondria, Miro1 is removed from mitochondria, a procedure that arrests organellar transport and facilitates mitochondrial clearance. Interestingly, a study using human fibroblasts and iPSC-derived dopaminergic neurons demonstrated that LRRK2 forms a complex with Miro1 as a precondition for its removal from damaged mitochondria, a process that is disrupted in LRRK2 G2019S PD patient models [119]. In this study, parameters of mitochondrial transport were not changed under basal conditions comparing LRRK2 G2019S mutant neurons with controls. However, treatment with the complex III inhibitor antimycin A, known to trigger mitochondrial depolarization and clearance, reduced mitochondrial motility and induced mitochondrial clearance in wild-type neurons, a process that was heavily delayed in *LRRK2* mutant neurons. Similarly, in a following study from the same group, baseline mitochondrial motility was unchanged between A53T α-syn mutant PD patient and control neurons, while antimycin A treatment evoked cessation of mitochondrial motility and subsequent mitophagy in wild-type neurons, both of which were delayed in *SNCA* mutant [120] and sporadic PD neurons [120,121]. Interestingly, pharmacological correction of Miro1 dynamics in both A53T α-syn mutant and sporadic PD iPSC neurons promoted clearance of damaged mitochondria and proved neuroprotective against mitochondrial stressors [121]. Dysregulation of Miro1 removal kinetics on mitochondria has emerged as a potential common mechanism shared by both sporadic and familial PD pathology, and may therefore represent a novel therapeutic target [122].

### 4.4. Mitophagy

Due to their post-mitotic state and high bioenergetic demand, neurons are particularly susceptible to mitochondrial dysfunction. Thus, mitochondrial quality control is essential for neuronal survival as defective mitochondria release ROS, eventually leading to neurodegeneration and cell death [123]. Dysfunctional mitochondria are removed via mitophagy, a selective form of autophagy.

In mammals, there are several pathways and proteins involved in mitophagy such as PINK1, Parkin, BNIP3L/NIX and FUNDC1 [124]. However, PINK1/Parkin-dependent mitophagy is the best characterized pathway for mitochondrial quality control [125,126,127,128]. Importantly, mutations in both *PINK1* and *Parkin* are known to cause early-onset forms of PD [129,130]. In this pathway, PINK1 acts as a mitochondrial stress sensor. Under physiological conditions, PINK1 is imported into mitochondria via the translocase of the outer membrane (TOM) and the translocase of the inner membrane (TIM), dependent on the mitochondrial membrane potential [131]. Once PINK1 reaches the IMM, it is immediately cleaved and degraded [132]. In defective mitochondria, however, the compromised membrane potential impairs mitochondrial import of PINK1. Consequently, full-length PINK1 accumulates on the OMM serving as an identifier for damaged mitochondria. PINK1 then phosphorylates ubiquitin molecules at serine 65 bound to OMM proteins [133,134,135] which, in turn, results in recruitment of the cytosolic E3 ubiquitin ligase Parkin to damaged mitochondria and its partial activation. Full activation of Parkin is achieved by phosphorylation at serine 65 via PINK1 [136,137]. Activated Parkin then preferentially uses phospho-ubiquitin to modify several proteins on the mitochondrial surface [138]. In this way, phosphorylated ubiquitin leads to further recruitment and activation of Parkin, creating a positive feedback loop [139,140]. Eventually, the damaged mitochondria are covered with phosphorylated ubiquitin chains, which then interact with autophagy receptors such as optineurin and nuclear dot protein 52 (NDP52). This leads to recruitment of autophagosomes and the delivery of damaged mitochondria to the lysosomes for degradation [141]. 

Most studies investigating PINK1/Parkin-mediated mitophagy in PD research have been performed in immortalized cell lines overexpressing PINK1 and/or Parkin. However, the existence and physiological relevance of this pathway in neurons has been a matter of debate [142]. Early investigations did not observe mitochondrial recruitment of overexpressed Parkin upon mitochondrial depolarization in primary mouse neurons [143], suggesting that bioenergetic differences between primary neurons and cultured cell lines may be responsible for the differences in Parkin translocation following mitochondrial depolarization. Subsequent studies, however, confirmed that primary rodent neurons are definitively able to undergo PINK1/Parkin-mediated mitophagy [144,145,146,147].

After having established that PINK1/Parkin-dependent mitophagy occurs in non-neuronal cells and primary rodent neurons, it remains to be elucidated whether this pathway is also relevant in human neurons. One report in human iPSC-derived dopaminergic neurons demonstrated that endogenous levels of PINK1 and Parkin are not sufficient to induce loss of all mitochondrial proteins upon loss of mitochondrial membrane potential [148]. However, this study did not directly investigate autophagosomal uptake or lysosomal degradation capacity, which may equally explain the lack of mitochondrial protein loss in iPSC neurons. Other investigations using the same cell type have shown that both endogenous [149] and overexpressed Parkin [150] are translocated to mitochondria following valinomycin-induced mitochondrial depolarization [150]. In this study, endogenous levels of PINK11 and Parkin were sufficient to lower the levels of mtDNA in response to valinomycin, consistent with a role for PINK1/Parkin in mitophagy in dopaminergic neurons [150]. Similarly, stabilization of endogenous full-length PINK1 and increased ubiquitin phosphorylation was observed [149,151]. Finally, absence of PINK1 in human iPSC-derived dopaminergic neurons leads to inhibition of ionophore-induced mitophagy, providing further evidence for the role of PINK1 in this form of mitophagy [99]. 

Mitophagy is impaired in many neurodegenerative diseases, leading to accumulation of defective mitochondria. Hence, human iPSC-derived neuronal models have greatly contributed to the study of mitophagic clearance in the pathogenesis of PD [152]. Several studies, outlined below, demonstrate impaired mitophagic flux in human iPSC-derived dopaminergic neurons carrying PD-related *PINK1* and *Parkin* mutations. *Parkin* mutant iPSC-derived dopaminergic neurons displayed impaired mitophagy using the mt-mKeima system that allows visualization of mitochondria fused with lysosomes [153]. Interestingly, Schwartzentruber and colleagues demonstrated that mitophagy levels are dependent on the cellular energetic status in *Parkin* mutant iPSC-derived neurons [154]. While *Parkin* mutant dopaminergic neurons depend on glycolysis early in differentiation and show increased mitophagy levels, neurons dependent on OXPHOS in the end stages of dopaminergic differentiation were clearly defective in basal and induced mitophagy.

In addition, iPSC studies using PD patient-derived neurons with *PINK1* mutation confirmed an impaired recruitment of overexpressed Parkin to mitochondria upon depolarization, which could be corrected via wild-type *PINK1* lentiviral expression [150]. Moreover, the kinase activity and levels of phosphorylated ubiquitin (Ser65) were significantly reduced in *PINK1* mutant iPSC-derived neurons [155,156]. However, PD-related *SNCA* and *LRRK2* mutations have also been shown to disrupt the PINK1/Parkin-mediated mitophagy pathway. Human iPSC-derived dopaminergic neurons containing either the A53T α-syn or the LRRK2 G2019S mutation displayed a stabilization of Miro1, thereby delaying the arrest of damaged mitochondria on microtubules and consequently their clearance via mitophagy [119,120]. Another study using LRRK2 G2019S iPSC-derived neurons reported higher mitochondrial motility and elevated bidirectional movement interfering with proper mitochondrial degradation processes [74]. This suggests that *LRRK2* mutations can actively prevent mitochondria from undergoing mitophagy. Furthermore, in iPSC-derived LRRK2 G2019S knock-in neurons, the pathogenic increase in LRRK2 kinase activity was elegantly correlated with malfunctions in autophagosome transport, potentially revealing a mechanism for impaired mitophagy in cells with fully functional PINK1 and Parkin [157]. Lastly, PD-related mutations in *GBA1* have also been associated with impaired mitophagy. Using *GBA1* mutant iPSC-derived dopaminergic neurons reduced expression of the mitophagy adaptor protein BNIP3L/NIX and diminished mitochondrial-lysosomal colocalization was reported [68]. Interestingly, a study using non-neuronal cells and fly models reported a physiological regulatory mechanism of PINK1/Parkin-mediated mitophagy by BNIP3 [158]. However, whether and how the interaction of PINK1/Parkin-dependent mitophagy pathway coordinates with other mitophagy pathways remains unclear. Additional investigations may help to understand how other mitophagy receptors including BNIP3L/NIX and FUNDC1 may possibly also compensate for defects in the PINK1/Parkin pathway.

### 4.5. Mitochondrial Calcium Handling 

Another important mitochondrial function is the regulation of intracellular Ca^2+^ needed for a multitude of cellular processes [159]. In neurons, the uptake of cytosolic or endoplasmic reticulum (ER)-stored Ca^2+^ into mitochondria regulates cellular Ca^2+^ homeostasis, prevents excitotoxicity, and facilitates neurotransmission [160,161]. Under physiological conditions, small Ca^2+^ oscillations are constitutively transferred from ER to mitochondria at contact sites termed mitochondria-associated ER membranes (MAMs), or present near voltage-gated calcium channels (VGCCs) in the synaptic terminal. Ca^2+^ enters the mitochondrial matrix through the mitochondrial Ca^2+^ uniporter (MCU) [162,163] where it is needed to maintain enzymatic function of the TCA cycle and subsequently ATP production [5]. This conversion of Ca^2+^ transients into ATP is especially crucial in the synaptic terminal, where mitochondria generate the fuel necessary for many aspects of neurotransmission. Mitochondria maintain their homeostasis through efflux of excess matrix Ca^2+^ via the mitochondrial permeability transition pore (mPTP), however, large Ca^2+^ transfers can lead to overload and subsequent opening of the mPTP, collapse of membrane potential, diminished OXPHOS and ATP production, elevated ROS formation, release of cytochrome c, and eventual cell death [164,165,166,167]. The ability of mitochondria to absorb, store, and release Ca^2+^ has been extensively reviewed elsewhere [168,169], therefore, the following section will focus on findings of mitochondrial Ca^2+^ dynamics in iPSC-derived neuronal models of PD. 

Given the importance of mitochondrial function in highly-energetic neurons, deregulated mitochondrial Ca^2+^ dynamics and neuronal distress are closely intertwined. In PD, the highly vulnerable dopaminergic neurons of the SNpc exhibit autonomous pacemaking activity accompanied by increased oscillations resulting from high presynaptic density of the L-type voltage-gated calcium channel Cav1.3 [170,171]. This causes continuous influx and increased cytosolic concentrations of Ca^2+^ which, when combined with the limited Ca^2+^-buffering capacity [172], has been proposed to contribute to their unique vulnerability in PD [172,173]. Importantly, selectively antagonizing L-type Ca^2+^ channels using isradipine has been proposed to be neuroprotective in iPSC-derived dopaminergic neurons from PD patients carrying heterozygous and homozygous mutations in *DJ-1*, a protein known to have a role in oxidative defense mechanisms [34]. In this study, the isradipine-mediated reduction of Ca^2+^ influx was sufficient to abolish the accumulation of neurotoxic oxidized dopamine, a precursor to neuromelanin build-up. In contrast, one group demonstrated that isradipine was not protective in iPSC-derived dopaminergic neurons from PD patients with *Parkin* mutations, showing instead that elevated expression and activity of T-type Ca^2+^ channels are more deleterious for this pathogenic genotype [174]. *Parkin* mutant iPSC-derived dopaminergic neurons present with increased association between ER and mitochondria, and subsequently increased mitochondrial uptake of ER-released Ca^2+^, as well as decreased cytosolic Ca^2+^ transients [175]. Conversely, iPSC-derived dopaminergic neurons from patients carrying *GBA1* mutations (N370S, L444P, and RecNcil) showed increased basal and caffeine-induced cytosolic Ca^2+^ levels, indicating impaired mitochondrial Ca^2+^ buffering and ER-mitochondrial Ca^2+^ transfer Additionally, LRRK2 G2019S PD patient neurons showed elevated intracellular Ca^2+^, possibly due to LRRK2 hyperactivity upregulating translation of Ca^2+^ signaling proteins [176]. Interestingly, these neurons also benefited from isradipine application, indicating that L-type Ca^2+^ channels are also dysregulated by LRRK2 overactivity.

Regulation of Ca^2+^ transfer from ER to mitochondria at MAM sites is critically important and is mediated by both ER- and mitochondrial-associated protein partners. One such ER-associated protein, vesicle-associated membrane protein-associated protein B (VAPB), was recently shown to bind α-syn [177]. Intriguingly, dopaminergic neurons from PD patients with *SNCA* triplication show high interference of α-syn with VAPB binding its partner on the OMM, resulting in disrupted MAM tethering, reduced Ca^2+^ transfer, and weakened mitochondrial ATP synthesis [177]. Interestingly, iPSC-derived dopaminergic neurons from *GBA1* PD patients (N370S mutation) exhibited reduced levels of neuronal calcium sensor-1 (NCS-1) [24], a Ca^2+^-binding protein which facilitates MAM formation and Ca^2+^ transfer into mitochondria [178]. This protein demonstrated neuroprotection against neurodegeneration in a toxin-induced mouse model of PD, which was associated with regulation of nigral dopaminergic Ca^2+^ dynamics [24]. This suggests that insufficient NCS-1 activity may impair mitochondrial Ca^2+^ handling and contribute to the unique SNpc vulnerability events in human neurons. Altogether, mishandled Ca^2+^ appears to represent a consistent pathological phenotype in PD iPSC-derived dopaminergic neurons, with different genetic backgrounds causing distinct mitochondrial alterations in Ca^2+^ regulation which contribute to their unique predisposed risk to neurodegeneration. 

Taken together, these findings demonstrate the value of patient-specific iPSCs for the delineation of disease- and cell type-specific mitochondrial Ca^2+^ handling pathologies including deregulated ER-associated channels and tethering machinery, synaptic terminal voltage-gated and Ca^2+^-permeable receptors, regulatory proteins and macromolecular complexes, and organellar membrane dynamics, all in a sophisticated model system with high translational relevance.

## 5. Conclusions

One of the greatest breakthroughs of regenerative medicine in this century was the discovery of iPSC technology in 2006 by Shinya Yamanaka and Kazutoshi Takahashi. Currently, human iPSC model systems are routinely used for disease modeling, deciphering cellular disease mechanisms, drug testing and screening, genetic engineering, and other valuable applications. In PD, patient-specific iPSC disease modeling has accelerated our understanding of the molecular mechanisms underlying dopamine neuron vulnerability and degeneration. The current lack of animal models capable of recapitulating human PD pathology in its entirety highlights the significant challenges in accurately defining the mechanisms underlying PD pathophysiology. PD-derived iPSC models are therefore a very powerful tool for examining disease mechanisms, defining novel targets, and developing effective therapeutic treatments in the future. Furthermore, advances in generating and differentiating iPSCs with greater consistency and reproducibility make iPSCs a promising cell source for tissue regeneration therapy. However, before iPSCs can be routinely used in clinical practice, their efficacy and safety need to be rigorously tested.

Mitochondrial dysfunction has been associated with neurodegeneration in PD for decades, but the precise role of mitochondrial perturbations in the vulnerability of dopaminergic neurons in PD remains undetermined. iPSC technology has broadened the understanding of mitochondria in health and disease, and therapies targeting mitochondrial dysfunction represent a promising potential tool to mitigate the damage to dopamine neurons in PD. Despite much progress in determining cell autonomous, disease-relevant phenotypes in human neurons (Table 1), more complex model systems with the inclusion of non-cell autonomous determinants of pathophysiology are needed to comprehensively define the molecular mechanisms underlying dopaminergic neuronal vulnerability.

In summary, iPSC disease modeling is an invaluable research tool to further understanding of disease progression and accelerate identification of relevant targets for drug development, which has immense potential to benefit large cohorts of patients affected by neurodegenerative diseases.

## Figures and Tables

**Table 1:** Literature on mitochondrial phenotypes described in iPSC-derived neuronal models of Parkinson’s disease.

Mitochondrial Phenotype	PD-Associated Gene	Mitochondrial Dysfunction in PD	References
Respiration and membrane integrity	*Parkin*	Deficits of ETC complex I activity	[67]
	*PINK1*		[83]
	*LRRK*		[74]
	*GBA1*		[68]
	*LRRK2*	Deficits of ETC complex III activity	[69]
	*Parkin*	Suppression of ETC complex IV proteins	[70]
	*SNCA*	Reduced respiratory capacity	[77,78,79]
	*DJ-1*		[34]
	*LRRK2*		[69]
	*VPS35*		[73]
	*GBA1*		[68]
	*SNCA*	Reduced membrane potential	[77,81]
	*PINK1*		[82,83]
	*LRRK2*		[75]
	*VPS35*		[73]
	*DJ-1*	Mitochondrial oxidant stress	[34]
Fusion, fission and morphology	*SNCA*	Abnormal mitochondrial morphology	[105]
	*Parkin*		[43,70,98,100,101]
	*PINK1*		[43]
	*LRRK2*		[75,102]
	*VPS35*		[73]
	*GBA1*		[68]
Axonal mitochondrial transport	*SNCA*	Altered mitochondrial motility	[118,120]
	*LRRK2*		[69,74,119]
	*Parkin*	Microtubule fragmentation	[115]
Mitophagy	*Parkin*	Impaired mitophagy/mitophagic flux	[153,154]
	*PINK1*		[99]
	*PINK1*	Reduction in kinase activity	[155,156]
	*LRRK2*	Malfunction in autophagosome transport	[157]
	*SNCA*	Delayed mitophagy through altered Miro1 kinetics	[120]
	*LRRK2*		[119]
	*PINK1*	Impaired Parkin recruitment	[150]
	*GBA1*	Diminished mitochondrial-lysosomal colocalization	[68]
Calcium handling	*LRRK2*	Increased cytosolic Ca^2+^ level	[32]
	*GBA1*		[176]
	*Parkin*	Increased association between ER and mitochondria	[175]
	*SNCA*	Disrupted MAM tethering	[177]
	*GBA1*	Reduced neuronal calcium sensor-1 (NCS-1) level	[178]

## Data Availability

Not applicable.
